# An iterative strategy to design 4-1BB agonist nanobodies de novo with generative AI models

**DOI:** 10.1038/s41598-025-10241-5

**Published:** 2025-07-14

**Authors:** Ivan Poddiakov, Dmitriy Umerenkov, Irina Shulcheva, Victoria Golovina, Vasilina Borisova, Irina Pozdnyakova-Filatova, Evgeniy Loktyushov, Galina Zubkova, Andrey Savchenko, Andrei Ulitin, Pavel Blinov

**Affiliations:** 1 Sber AI Lab, Moscow, 117997 Russia; 2 AIRI, Moscow, 123100 Russia; 3 BigBioBang LLC, Pushchino, Moscow Region 142290 Russia; 4 Pushchino Scientific Center for Biological Research of the Russian Academy of Sciences, Institute for Biological Instrumentation, Pushchino, Moscow Region 142290 Russia; 5 MIREA - Russian Technological University, Moscow, 119454 Russia; 6 Pushchino Scientific Center for Biological Research of the Russian Academy of Sciences, G.K. Skryabin Institute of Biochemistry and Physiology of Microorganisms, Pushchino, Moscow Region 142290 Russia; 7 Laboratory of Algorithms and Technologies for Network Analysis, HSE University, Nizhny Novgorod, 603155 Russia

**Keywords:** Drug discovery, Protein design

## Abstract

The 4-1BB receptor, a key member of the tumor necrosis factor receptor (TNFR) family, represents a highly promising target for cancer immunotherapy. In this study, we developed a novel *in silico* pipeline to design VHH domain antibodies targeting 4-1BB, leveraging knowledge-based amino acid distributions to generate optimized complementarity-determining region (CDR) sequences. Our computational approach progressively refined nanobody binding properties, yielding designs with binding scores comparable to or exceeding those of an established reference nanobody. From an initial set of 80 top-ranked *de novo* sequences, 65 were successfully assembled, with 35 validated by sequencing. Although this screening round did not yield a high-affinity binder *in vitro*, the results provide critical insights into the relationship between initial design parameters and successful genetic assembly. These findings highlight the potential of our pipeline while identifying key areas for further refinement, particularly in optimizing deep-learning models for antibody development. This work advances the broader effort to harness computational design for high-precision therapeutic antibody discovery.

## Introduction

Immunotherapy has gained significance as an effective and safe therapeutic strategy for various diseases, including cancer^1,2^. Among these therapies, antibodies are the predominant class of protein therapeutics, with over 160 antibody-based treatments currently approved worldwide. There is now a diverse range of antibodies, varying from full-sized molecules to smaller nanobodies.

In 1993, a team led by Raymond Hamers at the Vrije Universiteit Brussel discovered a unique type of antibody in camelids – the heavy chain-only antibody (HCAb) ^3^. This antibody differs structurally from conventional ones and inspired the development of a whole family of nanobodies. HCAbs consist solely of two heavy chains, each containing two constant regions (CH2 and CH3) and a variable region. This variable region, known as the variable domain of heavy-chain-only antibodies (VHH), can be recombinantly produced and has a molecular weight of approximately 15 kDa, which is about 10% of a conventional antibody, which makes the genes encoding their structure much simpler and cheaper to assemble, as well as accessible for expression in a simple *E. coli* system ^4^. VHH antibodies, or nanobodies, are able to provide a greater paratope diversity and maintain an interaction surface area comparable to classical antibodies  ^5^. Important advantages are the increased solubility and thermal stability of VHH^6,7^; VHH antibodies are easily “humanized”, camelid VHH domains have a higher degree of sequence similarity to human VH domains, reducing potential immunogenicity^8,9^.

Despite the significant interest from the pharmaceutical industry, developing therapeutic antibodies still relies on the immunization of animals or the screening of antibody libraries to identify candidates that bind to specific targets. These traditional methods can be labor-intensive, time-consuming, and may not always yield antibodies that interact with therapeutically relevant epitopes^10^. Computational approaches have emerged as powerful alternatives, yet most rely on existing antibody-antigen complex structures or sequence databases, which restrict their applicability to novel or poorly characterized targets^11^. There are successful examples of optimizing CDR sequences of an existing antibody-antigen receptor, which employed a fine-tuned ProteinMPNN model ^12^. IgDesign is the first antibody inverse-fold deep learning model to be successfully validated *in vitro*. It can generate antibodies with binding affinities equal to or higher than the reference antibodies ^13^. Several deep-learning approaches for protein design exist, with RFdiffusion successfully validated *in vitro*^14^. EAGLE is a diffusion-based model for de novo antibody design, which does not require any input structure with sequence recovery. It is capable of designing full antibody sequence conditioned on the antigen epitope structure, but their results lack experimental validation^15^. Villegas-Morcillo et al. ^16^ developed a similar approach for antibody sequence and structure co-design, utilizing desired properties (e.g., solubility and stability) as constraints for the antibody generation process. Luo et al. ^17^ showcased a custom diffusion-based model, which yielded highly competitive results in terms of protein design metrics, but it relies on an existing antibody-antigen complex.

In our study, we selected an important antigen as a target for the development of therapeutic antibodies. The 4-1BB receptor has garnered attention as a promising therapeutic target in cancer due to its broad expression profile and ability to stimulate various signaling pathways involved in generating a potent immune response. Since its discovery and demonstration of potential as a clinical target, significant progress has been made in studying 4-1BB and developing clinical therapeutics targeting it^18,19^. Its ability to modulate diverse immune cells – especially by mediating T-cell survival, proliferation, and effector function – makes it an attractive target ^20,21^. However, the first generation of agonistic monospecific 4-1BB antibodies to enter the clinic, urelumab (BMS-663513) and utomilumab (PF-05082566), failed due to hepatic toxicity or lack of efficacy, respectively^18,22^. Second-generation agonists have recently been developed and are based on selective activity only within the tumor microenvironment (TME) and on desired target cells, such as cytotoxic T-lymphocytes (CTLs), while minimizing off-target effects and toxicity. These agonists are bispecific antibodies designed to simultaneously bind to 4-1BB and a tumor antigen, such as Her2^23,24^, PD-L1^25–27^, FAP^28^, among others. These antibodies are expected to activate 4-1BB pathways within the TME selectively. Recent studies have demonstrated that the incorporation of a 4-1BB agonist nanobody into a 4-1BB/PD-L1 bispecific antibody (PM1003) results in significant therapeutic antitumor efficacy while exhibiting limited toxicity^29^.

We employed a minimalistic approach to the design of CDRs, which has demonstrated excellent results, as reported by Birtalan et al.^30^. We posited that contemporary machine learning algorithms utilized to compute specific binding proteins may more effectively derive affinity protein structures when using a limited set of amino acids in the antigen binding region instead of utilizing a more extensive composition of up to twenty amino acids. A database of natural antibody sequences has demonstrated that, although CDR sequences exhibit substantial diversity, there are distinct preferences for specific amino acids ^31–33^. Furthermore, structural databases indicate that these preferences become even more pronounced when residues that mediate antigen recognition through direct contacts are considered ^34–37^. Consequently, we selected VHH monodomain antibodies as the starting point for our calculations and experimental validation. This approach involved utilizing a minimalistic chemical composition of tyrosine and serine amino acids within CDR1, CDR2, and CDR3 to identify candidates that specifically bind to the 4-1BB receptor in humans. Additionally, drawing upon previous research focused on the synthesis and validation of synthetic antibody libraries characterized by both limited and natural diversity of CDR3 heavy chain composition^38,39^, we adopted an amino acid composition based on the following distribution: Tyrosine (20 percent), Serine (15 percent), Glycine (15 percent), and 16 other amino acids, excluding cysteine, each at a composition of 3.1 percent.

Starting our design, we identified suitable epitopes on the target surface. In conjunction with the 3D receptor structure and a VHH framework, these epitopes were then used as an input for the RFdiffusion model. This model generated backbone structures for the CDRs, which were subsequently fitted with an amino acid sequence using the ProteinMPNN model. All designs were evaluated using molecular modeling tools, and the top candidates underwent several rounds of sequence optimization using ProteinMPNN, aiming to improve their binding properties gradually. Then, we selected 80 candidates with the best interaction scores for laboratory validation. We employed an optimized approach for the laboratory screening of the selected candidates. This strategy comprised the following steps: the assembly of genetic constructs incorporating the gene for the predicted VHH, the expression of the resulting protein in the culture medium, and subsequent ELISA analysis. This screening methodology enabled us to effectively filter out protein variants with low affinity and those that were not expressed.

Our study aimed to leverage computational methods for nanobody development, followed by validation through a streamlined laboratory screening process. By integrating deep learning–driven protein generation and rapid experimental testing, we sought to establish a robust pipeline for de novo nanobody discovery. Importantly, this approach allowed us to systematically identify key challenges – such as reconciling in silico predictions with experimental expression yields. These insights provide a practical framework for researchers navigating similar interdisciplinary workflows. Ultimately, we demonstrate how an iterative computational approach can be incorporated into biological design while explicitly mapping bottlenecks to guide future optimizations in the field.

## Results

### CDR backbone and sequence design

We executed multiple iterations of candidate generation, yielding 18,000 candidate binders by the conclusion of Step 1, encompassing all design modalities (see Methods, First Step: CDR Generation). Our methodology utilized the existing 4-1BB-VHH complex (PDB: 7D4B^29^) as a reference point to match its binding properties.

**Figure 1:**
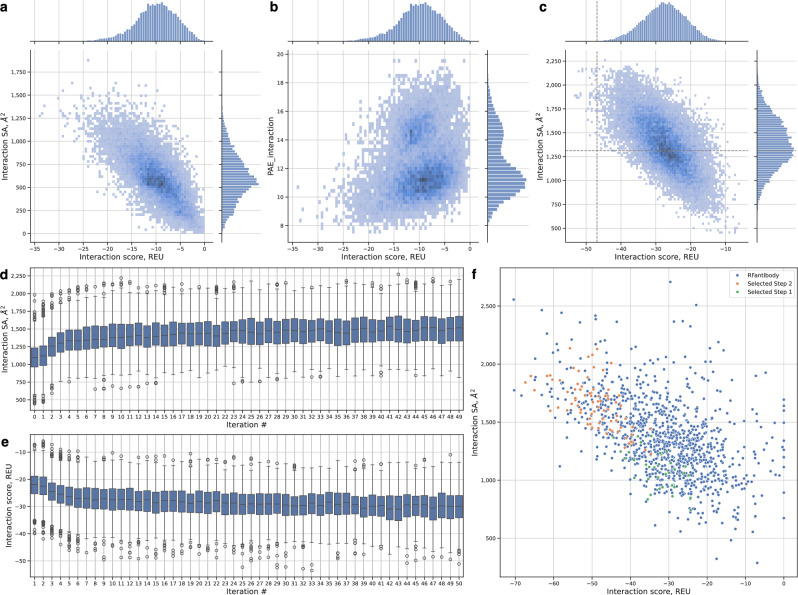
Computational scores of the designed candidate nanobodies. (**a**) Interaction score vs interaction SA of Step 1 designs. Histogram of co-distribution of Rosetta interaction score in REU (X-axis, lower is better) and interface SA (Y-axis, higher is better) of all Step 1 candidate nanobodies. (**b**) Distribution of interaction score and PAE_interaction metric of Step 1 designs. Histogram of co-distribution of Rosetta interaction score in REU (X-axis, lower is better) and PAE_intercation metric (Y-axis, lower is better) of all Step 1 candidate nanobodies. (**c**) Interaction score vs interaction SA of Step 2 designs. Histogram of co-distribution of Rosetta interaction score in REU (X-axis, lower is better) and interface SA (Y-axis, higher is better) of all Step 2 candidate nanobodies. Dashed line indicates scores of a reference VHH-4-1BB complex, -46.888 REU and 1313.2 Å$^2$, respectively. (**d**) Properties change over 50 iterations at design Step 2. The change of interaction SA (higher is better). Each individual boxplot illustrates the characteristics of the candidates generated during a particular iteration. (**e**) Properties change over 50 iterations at design Step 2. The change of Rosetta interaction score, REU (lower is better). (**f**) Our pipeline candidates compared to RFantibody. Point cloud of Rosetta interaction score in REU (X-axis, lower is better) and interface SA (Y-axis, higher is better). The blue dots represent 1000 RFantibody designs, the orange dots indicate 80 designs that were selected for experimental validation. The green dots represent 34 Step 1 designs, which served as initial candidates relative to the selected 80 and have been improved through iterative optimization.

We assessed the Rosetta interaction score, AlphaFold2 predicted aligned error of interacting protein chains (PAE_interaction) and interface surface area (SA) for each candidate binder. The interaction score distribution is illustrated in Fig. 1a, highlighting that the top candidates achieved scores as low as -34.179 Rosetta energy units (REU); nonetheless, this remained below the reference value of -46.888 REU for 7D4B. Furthermore, most candidates displayed lower interface surface areas; its distribution is featured in Fig. 1a. Only 0.5% of Step 1 designs achieved interface SA higher than the reference (1313.2 Å$^2$).

Figure 1b shows the distribution of PAE_interaction. For the transition to Step 2 of our pipeline, we selected only candidates with a PAE_interaction value lower than 10 and an interaction score lower than -20 REU. This selection process filtered out weakly interacting candidates while retaining a tractable number for further optimization, resulting in 160 initial candidates.

**Figure 2:**
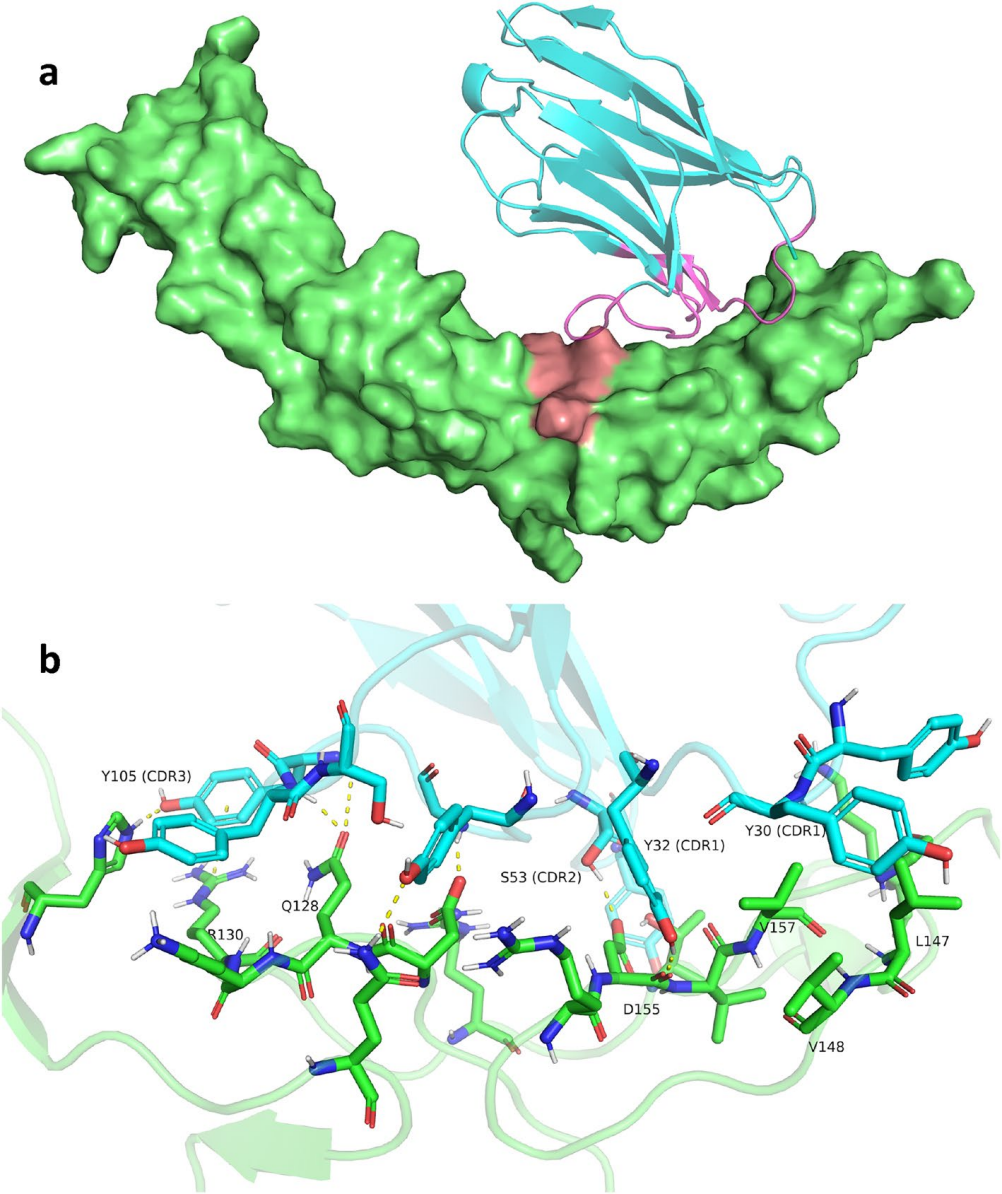
VHH_11 and 4-1BB interaction. (**a**) The candidate VHH_11 as predicted by ProteinMPNN following Step 2. The structure of VHH_11 (blue) interacts with 4-1BB (green), utilizing residues Q128, K129, and R130 as hotspots for RFdiffusion, which are highlighted in pink. All the CDR loops (magenta) interact with the target. (**b**) The predicted interactions of the CDR loops of VHH_11 with the receptor show that residues in each designed CDR are expected to form polar contacts with 4-1BB. Residue Y30 is predicted to engage in a nonpolar interaction with L147, V148, and V157 of the receptor.

Each candidate nanobody, selected following Step 1, underwent iterative optimization of the CDR sequences according to a specific procedure (see Methods, Second Step: Optimizing CDR sequence). The objective of this process was to enhance the interface SA. As illustrated in Fig. 1d, the changes in SA across iterations for each descendant of the selected designs were tracked. Consequently, the median interaction SA improved from 1098.0 to 1516.5, while the interaction score became lower, shifting from -21.969 REU to -29.853 REU (Fig. 1e), although the latter was not specifically targeted for optimization. Figure 1c shows the co-distribution of interface SA and interaction score compared to the reference complex; in Step 2, we identified 51 new designs that surpassed the interaction score of the reference 4-1BB-VHH complex. Given our limited resources for *in vitro* validation, we selected 80 candidates from a total of 749 variants. This selection includes the 51 new designs mentioned earlier. We specifically targeted candidates with interaction score values lower than -40 REU and prioritized descendants from as many initial Step 1 variants as possible. Amino acid sequences for 80 selected candidates are provided in Supplementary Data 1. The top design VHH_11, predicted by ProteinMPNN after Step 2, and its interaction with 4-1BB are illustrated in Fig. 2. The predicted binding pose of this candidate shows multiple interactions between the VHH and the receptor (Fig. 2b).

We assessed the similarity of CDR loops in each candidate selected for *in vitro* validation against the CDR loops found in the PDB. The sequences were queried using blastp^40^. For each candidate sequence, we identified the top blast hit and calculated the similarity between its CDRs and those of the candidates (Supplementary Fig. 1). The resulting similarity was relatively low, averaging below 50%.

### Our approach compared against RFantibody

We conducted a retrospective benchmarking study against the recently published RFantibody method ^41^ to validate our computational pipeline and clarify its relative performance. RFantibody employs a computational strategy similar to ours, involving an RFdiffusion model optimized explicitly for antibody design and a ProteinMPNN model. However, RFantibody was publicly released over a year after we initially generated our nanobody candidates using the original RFdiffusion model.

We generated approximately 1,000 RFantibody candidates targeting the identical epitope on 4-1BB used in our study. These candidates were evaluated using Rosetta interaction scores and interface SA. The comparative analysis showed that RFantibody initial results were superior to those of our original unoptimized candidates generated using vanilla RFdiffusion. However, after iterative optimization, our candidates matched the best of RFantibody-generated results, demonstrating the effectiveness of our iterative refinement process (Fig. 1f).

This comparison underscores that while RFantibody provides strong initial performance out-of-the-box, further optimization of RFantibody-generated designs using our iterative strategy may lead to even greater improvements in predicted antibody-antigen binding characteristics. Such integrated approaches could represent a promising direction for future antibody design strategies leveraging generative AI models.

### Construction of the VHH gene and the pET22b-VHH vector

PCR of the plasmid vector was performed using specific primers containing the restriction site sequence specific to the BsaI restriction enzyme. The optimized PCR conditions were as follows: 95$^{\circ }$C for 5 minutes (1 cycle); 95$^{\circ }$C for 30 seconds, 60$^{\circ }$C for 30 seconds, and 72$^{\circ }$C for 3 minutes (25 cycles), followed by 72$^{\circ }$C for 3 minutes (1 cycle). Amplification was carried out using a reaction mixture containing the high-fidelity polymerase Blitz (BelBioLab, Republic of Belarus). The PCR mixture containing the linear PCR product of the plasmid was subsequently purified using the CleanUp kit (Evrogen, Moscow).

The purified plasmid was subjected to restriction digestion using the BsaI (NEB, UK) and DpnI (NEB, UK) enzymes or only DpnI (NEB, UK) for ligase-free cloning. Electropherogram of the pET22b Plasmid Vector in a 1% Agarose is reported in Supplementary Fig. 2.

Gene synthesis was performed via PCR using annealing and amplifying overlapping oligonucleotides in multiple stages. A typical electropherogram of PCR products is reported in Supplementary Figure 3. The optimized PCR conditions for assembling gene fragments were as follows: 95$^{\circ }$C for 5 minutes (1 cycle); 95$^{\circ }$C for 15 seconds, 60$^{\circ }$C for 10 seconds, and 72$^{\circ }$C for 30 seconds (10 cycles); followed by 72$^{\circ }$C for 90 seconds (1 cycle). The optimized PCR conditions for amplifying the full-length gene sequence were 95$^{\circ }$C for 5 minutes (1 cycle), 95$^{\circ }$C for 15 seconds, 60$^{\circ }$C for 10 seconds, and 72$^{\circ }$C for 60 seconds (10 cycles), followed by 72$^{\circ }$C for 90 seconds (1 cycle).

PCR products were analyzed via electrophoresis in a 2% agarose gel. As a result of the study, 65 out of the planned 80 genes were successfully assembled, and 35 were validated by sequencing.

**Figure 3:**
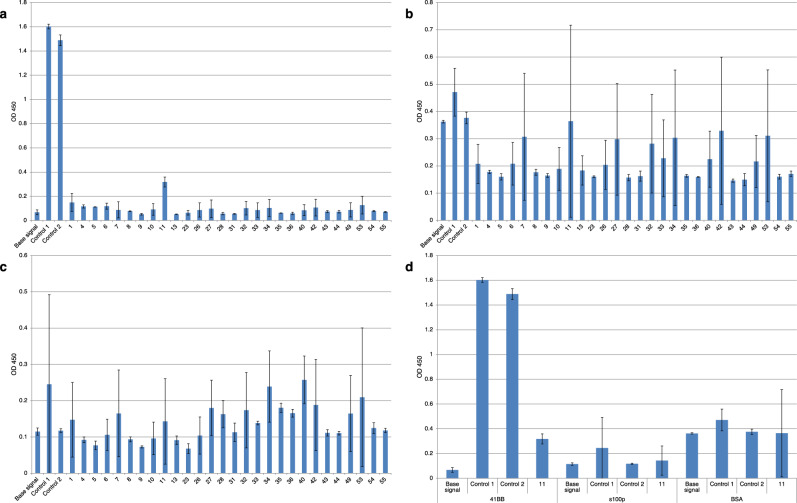
ELISA analysis of the designed nanobodies. X-axis represents the index numbers of VHH clones under study, culture fluid of the clone-producer after expression was used for analysis. “Base signal” – signal of all ELISA components in the absence of VHH; “Control 1” – culture media after expression of the clone-producer of the control VHH antibody 7D4B; “Control 2” – purified VHH antibody 7D4B. “11” is the culture media after expression of the culture clone of the in silico calculated VHH_11. All experiments were performed in 2 technical repetitions, column top – mean value, error bars – confidence interval. (**a**) The level of the designed nanobodies binding to 4-BB antigen. (**b**) The level of the designed nanobodies binding to BSA protein. (**c**) The level of the designed nanobodies binding to S100P protein. (**d**) Comparison of signals for target and non-target antigens for control VHH 7D4B and VHH_11. While VHH_11 achieves the highest binding signal among our designs, this signal level is equivalent to the average affinity to BSA.

### Expression of VHH

All plasmids validated through sequencing were used to transform *E. coli BL21 (DE3)* cells. Protein expression was induced under optimized conditions by adding 0.5 mM IPTG at 20$^{\circ }$C for 16 hours. Following expression, the culture medium was separated from the cells by centrifuging and used for ELISA and detecting secreted protein via electrophoresis under denaturing conditions. Electropherogram in 20% PAAG of the culture media analysis after expression is reported in Supplementary Figure 4. Based on the results, only 10 clones exhibited a visible protein band on the gel, with productivity ranging from 0.1 to 20 mg of protein per liter of culture.

**Figure 4:**
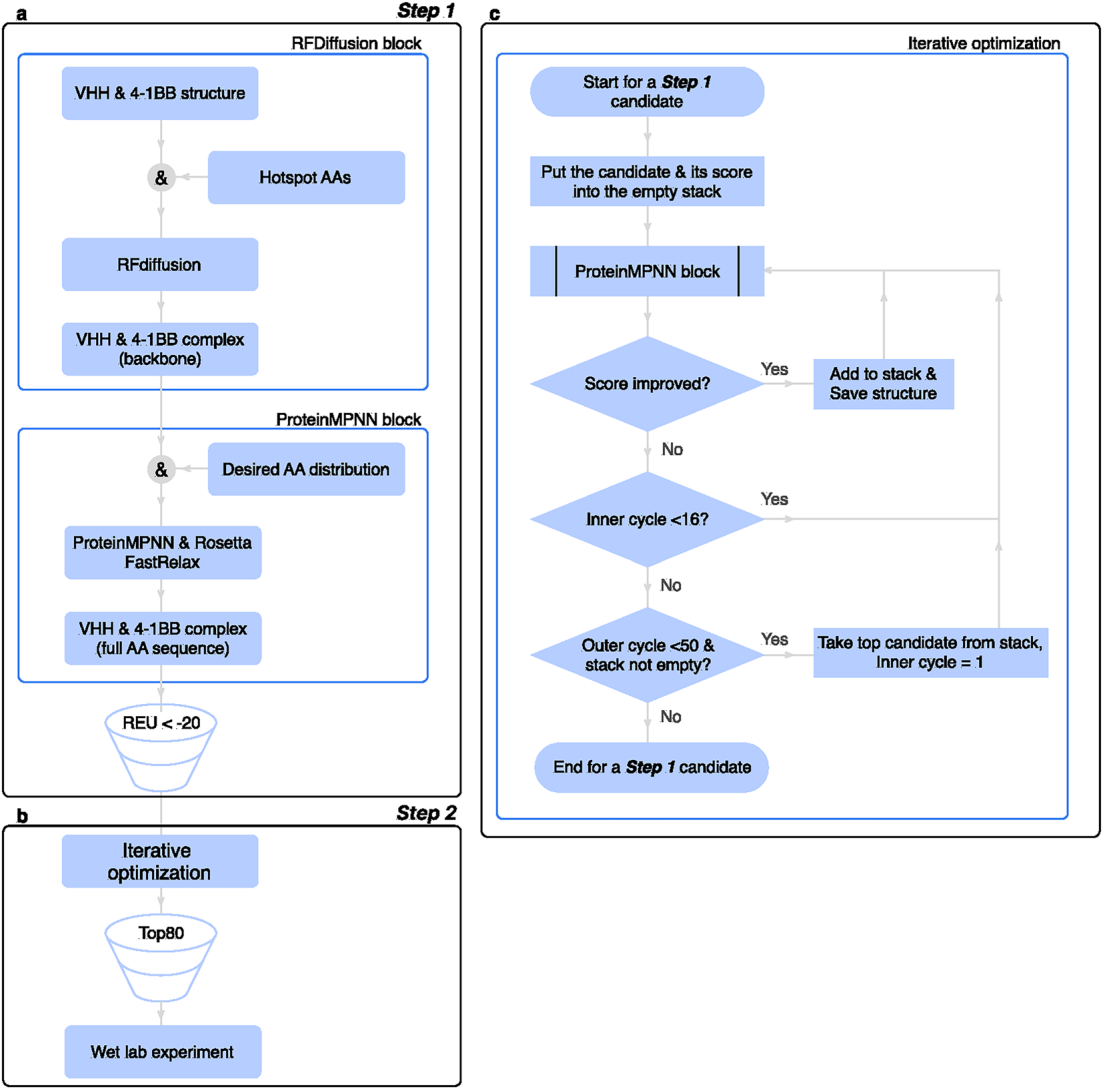
Proposed generative pipeline. (**a**) Step 1 of the pipeline comprises an RFdiffusion block and a ProteinMPNN block. Initial structure and sequence are generated in this step. (**b**) Step 2 of the pipeline. Every Step 1 candidate undergoes an iterative optimization procedure, and top-scoring candidates undergo laboratory testing. (**c**) Detailed algorithm of the iterative optimization.

### Binding specificity assessment with ELISA

To determine the binding specificity of VHH antibodies, the target antigen 4-1BB was adsorbed onto the surface of an immunological plate. The calcium-binding protein S100P and bovine serum albumin (BSA) were also adsorbed to assess cross-reactivity. As a positive control, for which literature data on binding to the 4-1BB antigen exists, we used VHH 7D4B^29^. To reduce nonspecific signal levels, culture medium samples were pre-incubated with 1% dry milk containing various proteins.

ELISA results aligned with prior electrophoretic analysis, confirming that most clones had low expression levels (Fig. 3a). None showed detectable binding to 4-1BB and acceptable expression levels. All tested VHH constructs exhibited high nonspecific binding to BSA (attributed to the structural properties of albumin family proteins; Fig. 3b) and moderate binding to S100P (Fig. 3c). This cross-reactivity suggested insufficient specificity for further investigation. A single clone, VHH 11, demonstrated weak binding to 4-1BB (Fig. 3d) with the average signal higher than the base signal. However, its signal intensity was 10 times lower than the positive control’s. Based on the above, we did not select any clone for a further characterization of the dissociation constants of the antigen-nanobody complex.

## Discussion

We identified several nanobody candidates featuring unique sequences and predicted binding properties that aligned with the reference properties. However, this alignment does not necessarily guarantee the successful identification of robust specific binders. We cannot definitively prove that certain generative methods are unsuitable for this task, nor was that our intention. However, it has been noted that AlphaFold2^42^ performs less effectively with antibody CDRs^43^. Additionally, it has been observed that vanilla RFdiffusion, which excels at designing binders with standard secondary structures, experiences a decline in performance when modeling CDR sequences^41^, which typically exhibit unique secondary structures. Therefore, it is essential to consider and develop other specialized tools for in silico antibody folding and evaluation rather than relying solely on a universal pipeline for protein structures. With the fine-tuned RFdiffusion model introduced as part of the RFantibody framework, a potential area of research could involve integrating our iterative strategy with antibody-finetuned models.

To address the objectives, the laboratory experiments used the following strategy: we focused on the assembly and cloning of 80 constructs containing VHH genes for individual extracellular expression. Cytoplasmic expression of nanobodies in *E. coli* poses challenges due to the presence of at least one conserved disulfide bond. The reducing environment of the cytoplasm can disrupt disulfide bonds, compromising the stabilization of the secondary structure and increasing the risk of insolubility due to unfavorable interactions among folding intermediates, ultimately leading to protein aggregation^44,45^.

Compared to intracellular expression, extracellular protein production offers several advantages. First, extracellular expression can prevent protein aggregation and inclusion body formation. Second, the secretion of the protein from the cytoplasm to the periplasm provides an oxidative environment that promotes proper protein folding and disulfide bond formation^46^, which is essential for its biological activity. Moreover, the secretion of proteins into the culture medium eliminates the need for cell lysis and downstream purification steps, simplifying the bioprocess and significantly improving product purity by avoiding intracellular contamination^47^. Consequently, various strategies have been implemented to achieve extracellular expression ^48^.

Based on the previous studies, we selected an optimized expression system for secretion into the culture medium using the pelB leader sequence ^4^. Despite these optimizations, no clones with high productivity were obtained; only one clone yielded a high protein output of approximately 20 mg/L. This result suggests that the chosen strategy for designing the variable amino acid region may have adversely affected the final protein fold. Studies indicate a significant role of CDR3 in the stability of the final VHH structure^49^. In our study, we selected a strategy maximizing CDR3 variability unique to each candidate. While our algorithmic approach successfully maximized CDR3 variability, further refinements may be needed to ensure optimal folding of VHH-type structures.

To assess the affinity of the synthesized nanobodies, we employed a widely used enzyme-linked immunosorbent assay (ELISA) on culture supernatants. This method allows for evaluating binding levels without additional purification while determining whether the expression is present in the analyzed samples^50^. The analysis indicated that most clones did not yield sufficient protein for evaluation, and ELISA results were negative. Among the clones with detectable protein production via electrophoresis, none exhibited specific binding in the ELISA, suggesting a need for further optimization of antigen recognition.

Of the 80 VHH sequences designed de novo without prior information on epitope-binding VHH antibodies, 35 were validated through sequencing, and 65 were successfully assembled. The low accuracy of the synthesized genes may be attributed to the selection of variable CDR sequences, which contained serine (S)- and tyrosine (Y)-rich patterns. This resulted in DNA instability due to the limited number of codons available for encoding these amino acids. Consequently, this instability may have increased the error rate during oligonucleotide synthesis and contributed to recombination events when the assembled *in vitro* plasmid was introduced into cells. Our chosen approach to the amino acid composition of CDRs has previously yielded good results. The effectiveness of minimalist design was corroborated in a separate study^30^. Researchers synthesized and examined libraries of human Fab fragments with a minimalist Tyr/Ser composition in HCDR1 and HCDR2 while incorporating a broader amino acid composition in HCDR3, including Tyr, Ser, Gly, and Arg. Selection experiments yielded high-affinity antibodies against two human antigens, VEGF and HER2. Notably, no correlation was observed between nonspecific binding and the content of Tyr, Ser, or Gly in the antigen-binding site. However, a clear correlation was found between the presence of Arg and increased nonspecific binding. Since antibody affinity is determined by the CDR regions, it is possible that the “depletion” of CDR1 and CDR2, which were saturated with tyrosine and serine residues, was not the most effective strategy. Given that CDR1 and CDR2 are already spatially constrained by germline-encoded sequences, a more refined approach is required for optimizing their design. Additionally, expanding the amino acid composition beyond the selected residues to include natural diversity may be necessary. Lowering Arg content in the antigen-binding site in future experiments may result in more specific binders.

The selected starting amino acid distribution for CDRs likely plays a key role in influencing the outcomes of genetic construction building, offering valuable insights for optimization. Moreover, our findings support important evidence that vanilla RFdiffusion may not be the optimal approach for CDR sequence design, highlighting opportunities for further research.

## Limitations of the study

Due to limited resources, we have validated only a small number of candidates and produced designs only with restricted amino acid content. Furthermore, we have not subjected the top candidates from Step 1 to in vitro validation. As a result, we cannot assess the impact of iterative optimization on the actual binding affinities.

## Methods

### Starting VHH sequence and structure preparation

We decided to use a constant sequence derived from the human DP47 germline ^51,52^ and an FR2 composition of F37/G44/T45/ F47 ^53,54^ in our designs. Subsequent in silico modeling of VHH sequences was conducted by varying seven amino acid (27-33 Kabat numbering) positions in CDR1 (restricted to Tyr or Ser), seven positions (52a-57 Kabat numbering) in CDR2 (also restricted to Tyr or Ser), and 10-12 positions (95-99 Kabat numbering) in CDR3^55^, incorporating all amino acids except Cys and Met, or restricted to Tyr, Ser and Gly (see Table 1).

**Table 1:** Desired amino-acid content in CDR sequences.

Design mode	CDR 1-2		CDR 3	
	Used aa	Omitted aa	Used aa	Omitted aa
cdr123-min	Tyr (50%), Ser (50%)	Other 18	Tyr (50%), Ser (25%), Gly (25%)	Other 17
cdr12-min-3-germ	Tyr (50%), Ser (50%)	Other 18	Tyr (20%), Ser (15%), Gly (15%), Other (3.3% each)	Cys, Met

We selected seed protein models from 3EAK ^8^ and 7D4B ^29^ 3D structures for the nanobody design. The former contains a VHH domain and resembles the sequence with minimal immunogenicity described earlier. The latter structure provides a model for the human 4-1BB receptor. Then, the nanobody structure was modified with the Rosetta Remodel protocol^56^ to change the FRs and CDRs sequences according to the minimal immunogenicity variant and DP47 VH germline human, respectively.

Several epitopes for nanobody-receptor binding were chosen so that the 4-1BBL binding site was not overlapped^57^, as well as residue C121, which participates in 4-1BB dimerization^58^, and residues N138 and N149, which undergo post-translational modification^21^. These epitopes were selected using epitope prediction tool SEMA^59^. We chose five appropriate epitopes, each defined by three 4-1BB residues for further CDR backbone design (Table 2). The structure of 4-1BB, including its structural features and selected epitopes, is shown in Supplementary Figure 5.

**Table 2:** Selected 4-1BB epitopes.

Epitope label	4-1BB residues
1	Q128, K129, R130
2	S80, T81, S82
3	D155, V156, V157
4	K107, Q108, E153
5	R73, P90, K107

Each epitope is defined by three amino acid residues, which are subsequently used as input to the RFdiffusion model. Residue numbers are provided according to UniProt Q07011 indexing.

### First step: CDR generation

CDR generation for the nanobody structure was carried out in two steps (Fig. 4). The first step consists of a Diffusion block and an MPNN block (Fig. 4a). The modified VHH structure and 4-1BB structure were stored in one pdb file. The CDR backbone was designed with an RFdiffusion model in the context of the receptor^14^. Residue numbers for each selected epitope were provided as script parameters. The generation process involved fixing residues (Kabat numbering) 1–26, 34–52, 58–95, and 100–113 from chain A and target chain B. In this setup, sequences of 7 amino acids were generated to replace the CDR1 and CDR2 regions, while a backbone sequence of 6–12 amino acids could be positioned in the CDR3 region. These regions were generated through partial diffusion, utilizing fixed residues as a framework. The diffusion process lasted 50 steps, employing the default model and diffuser parameters provided in the checkpoint Complex_base_ckpt.pt (https://github.com/RosettaCommons/RFdiffusion). Typically, we generated 200 backbones for each design attempt.

Next, the following procedure generates a sequence for each generated backbone composed of GLY residues. This involves two rounds of sequence generation using ProteinMPNN^60^, followed by relaxation using Rosetta FastRelax^61^. We created one sequence for each backbone structure individually, with residues similar to the diffusion being fixed and amino acid content biased, as described in Table 1. These restrictions include the desired rates of amino acid residues and specified prohibited residues.

Then, each prediction from the final round is validated using AlphaFold2^42^. The PAE_interaction metric is used to determine the reliability of the interaction and folding^62^. Of all the metrics used to assess the quality of the AlphaFold2 reconstruction, the authors of the method consider the PAE_interaction metric to have the highest predictive power, and designs are more likely to be successfully validated if this metric is less than 10. We adhere to the evaluation using AlphaFold2 as outlined in the work by Bennett et al. ^62^(https://github.com/nrbennet/dl_binder_design).

Protein-protein interface scoring was performed in Rosetta for each generated structure in complex with the receptor. Initially, the structure was relaxed using Rosetta Relax^63,64^, with a constraint on the movement of the backbone and centroid. Following this, the interaction energy was calculated. Complexes with an interaction score below -20 REU were selected for further refinement in the second stage of the design process. This threshold effectively filtered out weakly interacting candidates while retaining a tractable number for further optimization.

### Second step: optimizing CDR sequence

At the second step of the design (Fig. 4b), an iterative improvement of the binding parameters of the nanobody to the 4-1BB receptor was carried out.

Figure 4c describes the iterative optimization procedure implemented in our study. Candidate optimization was carried out in several cycles. The CDR sequences of each candidate molecule were re-generated by ProteinMPNN, after which structures with altered sequences were minimized by Rosetta FastRelax. If the surface area of the nanobody-receptor contact in the variant increased relative to its predecessor, then this variant was added to the stack sorted by the value of the SA contact. In total, 16 attempts to improve the score are made. Next, the inner cycle is repeated up to 50 times, optimizing the sequence of the top rank in the stack. CDR sequences were also generated in two modes with similar fixed residues and different restrictions on the amino acid residues of the generated chain regions.

The contact surface area was estimated using the following formula: $SA_{interface} = SA_{nanobody} + SA_{4-1BB} - SA_{complex}$, using Shrake–Rupley algorithm^65^. Incorporating interface areas is critical for discriminating binders in machine learning applications^66^.

We additionally discarded VHH designs, which have more than five FR residues in close proximity to receptor atoms. We considered a residue in contact with the other chain if any of its atoms are within 4.0 Å of any receptor atom. We also imposed a constraint such that at least two CDRs are in contact with the receptor. For CDR3 residues, contact is mandatory. Scoring of a protein-protein interaction is carried out in Rosetta for each structure obtained at the second step, similarly to the first step. Candidate molecules rated below -40 REU were selected for further validation in a laboratory experiment.

### Comparison against RFantibody

We followed the official installation and usage instructions provided at https://github.com/RosettaCommons/RFantibody. Additionally, we fixed the residues (using Kabat numbering) 1–26, 34–52, 58–95, and 100–113 from chain A while targeting chain B. Sequences of 7 amino acids were generated to replace the CDR1 and CDR2 regions, and a backbone sequence of 6–12 amino acids was positioned in the CDR3 region, similar to the design approach we implemented in our work. However, we did not filter out any RFantibody designs based on predicted contacts or amino acid distribution.

Using RFantibody, we made a single design campaign, resulting in 1,000 designs. To compare our designs against those from RFantibody, we removed a Rosetta Relax constraint on the movement of the backbone, as it prevented several RFantibody designs from relaxing fully. After this adjustment, we reevaluated our 80 selected candidates and the initial Step 1 candidates using the updated relaxation parameter for a correct comparison.

### Bacterial strains, plasmid, and media

The VHH gene was cloned using pET22b vectors (Invitrogen, USA). The *E. coli* strains *E. Cloni* and *BL21 (DE3)* were used as host strains for recombinant plasmids. Bacteria were cultured in 2 YT medium. pET22b was used as an expression vector.

### Construction of the VHH gene

The VHH gene was cloned using synthetic oligonucleotides with overlapping sites by assembly PCR using Blitz DNA polymerase (BelBioLab, Russia). The VHH gene was amplified in two steps. Next, it was purified using the Cleanup kit (Evrogen, Moscow) and treated with T7 endonuclease (NEB, England). The correct part of the gene was then spliced using PCR.

### Construction of the pET22b-VHH vector

Enzyme restriction sites BsaI were introduced into the pET22b vector (Novagen, USA) using inverse PCR. The assembled pET22b-VHH vector was amplified using specific primers pelB-pET22b_rev(30-mer) TCT TTT GGT CTC TGG CCA TCG CCG GCT GG, 6his-pET22b_for(38-mer) TCT TTT GGT CTC TCA CCA CCA CCA CCA CCA CTG AGA TC using inverse PCR for further ligase-free cloning. The amplified pET22b vector with restriction sites and VHH gene were digested with the restriction enzymes BsaI (Neb, England). DNA after restriction was prepared using the Cleanup kit (Evrogen, Moscow). Ligation was carried out in a molar ratio of 1 (vector): 5 (gene) in 5 µl of the reaction mixture and incubated for 20 h at a temperature of 4$^{\circ }$C. The recombinant pET22b-VHH vector was transformed into *E. coli* cells (*E. cloni* strain) using electroporation and plated on Petri dishes with agar containing ampicillin (10 µg/ml). The colonies were checked for the presence of an insert containing the VHH gene by PCR using the flanking primers indicated above. Plasmid DNA was isolated using the PlasmidMiniPrep Kit (Evrogen, Moscow). The correspondence of the nucleotide sequence of the gene was confirmed by DNA sequencing (Evrogen, Moscow). The kit ClonExpress II One Step Cloning Kit (Vazyme, China) was used for ligase-free cloning according to the manufacturer’s instructions. The map of the pET22b-VHH vector is provided in Supplementary Figure 6.

### Expression of VHH

The recombinant plasmid pET22b-VHH was transformed into the *E. coli BL21 (DE3)* strain by electroporation. The successfully transformed *E. coli BL21 (DE3)* cells containing recombinant plasmid were inoculated into 1 ml of 2 YT supplemented with 10 µg/ml ampicillin and shaken at 210 rpm/min at 37 $^{\circ }$C overnight. An overnight culture of 50 mkl was inoculated into 1 ml of fresh 2 YT medium with ampicillin at a shaking speed of 210 rpm/min at 37 $^{\circ }$C until the OD600 value of the culture reached 0.6. The VHH gene was induced to express the recombinant protein by IPTG in a final concentration of 0.5 mM for 12 h at 20 $^{\circ }$C. The culture medium after expression was separated from the cells by centrifuging and used for ELISA. The recombinant protein secreted into the culture fluid was precipitated using Trichloroacetic Acid. The protein fraction was dissolved in a sample buffer and analyzed by denaturing gel electrophoresis.

### Electrophoresis

Using established techniques, the recombinant protein secreted into the culture fluid was resolved by denaturing discontinuous SDS–PAGE in 20% gels. Proteins were visualized in the gels by staining with Coomassie Blue G250. Prestained size markers were used as standards.

### ELISA

96-well plates with a well volume of 0.2 mL were used for analysis. After each reaction component, the wells were washed with PBST. 50 µl of 4-1BB solution in carbonate buffer (concentration 0.5 µg/mL) was applied to the plates and incubated at 37 $^{\circ }$C for 1 h. Free binding sites were blocked with milk powder solution (1%) for 1 hour at 37 $^{\circ }$C. A sample of culture fluid containing target protein was mixed in equal proportion with 2% milk powder and applied to the plastic of immunoplate, then incubated for 1 hour at 37 $^{\circ }$C. After incubation, a solution of 9E10 secondary antibody specific to myc-tag, which is part of the fusion protein, was applied to the wells and incubated under the same conditions. Next, a solution of antibody-anti-M13-HRPO peroxidase conjugate antibody (PBST) was applied to the wells for one h at 37 $^{\circ }$C. In the last step, 100 µl of TMB solution was added to the washed wells one at a time. The reaction of peroxidase with substrate was stopped by adding an equal volume of 10% sulfuric acid. The result was evaluated by color intensity using a flatbed photometer at a wavelength of 450 nm.

### Statistics and reproducibility

The generation of sequences and structures was performed independently for each variant, ensuring that no variants were discarded or disproportionately represented at any point during the design process. A summary table detailing key computational parameters of our pipeline is provided as Supplementary Table 1. Two assemblies were sequenced for each VHH clone. Based on these data, the percentage of correct clones was calculated. All ELISA experiments were conducted in two technical replicates. These results represent the mean value along with the confidence interval.

## Supplementary Information

Supplementary Information 1.

Supplementary Information 2.

## Data Availability

This study did not generate new materials. Any additional information required to reanalyze the data reported in this paper is available from the corresponding author upon request.

## References

[1] Kciuk, M. et al. Recent advances in molecular mechanisms of cancer immunotherapy. *Cancers* **15**, 2721 (2023). 37345057 10.3390/cancers15102721 PMC10216302

[2] Zhao, X. & Starr, T. * & Subramanian, S* (From molecular mechanisms to clinical applications, Advancing cancer immunotherapy, 2023). 10.3390/cancers15164197 PMC10453472 37627225

[3] Hamers-Casterman, C. et al. Naturally occurring antibodies devoid of light chains. *Nature* **363**, 446–448 (1993). 8502296 10.1038/363446a0

[4] Chao, S. et al. Highly expressed soluble recombinant anti-gfp vhhs in escherichia coli via optimized signal peptides, strains, and inducers. *Front. Mol. Biosci.* **9**, 848829 (2022). 35359590 10.3389/fmolb.2022.848829 PMC8960375

[5] Mitchell, L. S. & Colwell, L. J. Analysis of nanobody paratopes reveals greater diversity than classical antibodies. *Protein Eng. Des. Sel.* **31**, 267–275 (2018). 30053276 10.1093/protein/gzy017 PMC6277174

[6] Van der Linden, R. et al. Comparison of physical chemical properties of llama vhh antibody fragments and mouse monoclonal antibodies. *Biochim. Biophys. Acta (BBA)-Protein Struct. Mol. Enzymol.* **1431**, 37–46 (1999). 10.1016/s0167-4838(99)00030-8 10209277

[7] Ladenson, R. C., Crimmins, D. L., Landt, Y. & Ladenson, J. H. Isolation and characterization of a thermally stable recombinant anti-caffeine heavy-chain antibody fragment. *Anal. Chem.* **78**, 4501–4508 (2006). 16808459 10.1021/ac058044j

[8] Vincke, C. et al. General strategy to humanize a camelid single-domain antibody and identification of a universal humanized nanobody scaffold. *J. Biol. Chem.* **284**, 3273–3284 (2009). 19010777 10.1074/jbc.M806889200

[9] Rossotti, M. A., Bélanger, K., Henry, K. A. & Tanha, J. Immunogenicity and humanization of single-domain antibodies. *FEBS J.* **289**, 4304–4327 (2022). 33751827 10.1111/febs.15809

[10] Wilson, P. C. & Andrews, S. F. Tools to therapeutically harness the human antibody response. *Nat. Rev. Immunol.* **12**, 709–719 (2012). 23007571 10.1038/nri3285 PMC7097371

[11] He, X.-h. et al. Ai-driven antibody design with generative diffusion models: current insights and future directions. *Acta Pharmacol. Sin.* 1–10 (2024). 10.1038/s41401-024-01380-y PMC11845702 39349764

[12] Dreyer, F. A., Cutting, D., Schneider, C., Kenlay, H. & Deane, C. M. Inverse folding for antibody sequence design using deep learning. 10.48550/arXiv.2310.19513 (2023).

[13] Shanehsazzadeh, A. et al. *In vitro validated antibody design against multiple therapeutic antigens using generative inverse folding* (NeurIPS, In Generative AI and Biology (GenBio) Workshop, 2023).

[14] Watson, J. L. et al. De novo design of protein structure and function with rfdiffusion. *Nature* **620**, 1089–1100 (2023). 37433327 10.1038/s41586-023-06415-8 PMC10468394

[15] Cohen, T. & Schneidman-Duhovny, D. Epitope-specific antibody design using diffusion models on the latent space of esm embeddings. In *ICLR 2024 Workshop on Generative and Experimental Perspectives for Biomolecular Design* (2023).

[16] Villegas-Morcillo, A., Weber, J. M. & Reinders, M. J. Guiding diffusion models for antibody sequence and structure co-design with developability properties. *PRX Life* **2**, 033012 (2024).

[17] Luo, S. et al. Antigen-specific antibody design and optimization with diffusion-based generative models for protein structures. *Adv. Neural. Inf. Process. Syst.* **35**, 9754–9767 (2022).

[18] Chester, C., Sanmamed, M. F., Wang, J. & Melero, I. Immunotherapy targeting 4–1bb: mechanistic rationale, clinical results, and future strategies. *Blood J. Am. Soc. Hematol.* **131**, 49–57 (2018). 10.1182/blood-2017-06-741041 29118009

[19] Claus, C., Ferrara-Koller, C. & Klein, C. The emerging landscape of novel 4-1bb (cd137) agonistic drugs for cancer immunotherapy. In *MAbs*, vol. 15, 2167189 (Taylor & Francis, 2023). 10.1080/19420862.2023.2167189 PMC9897756 36727218

[20] Vinay, D. S. & Kwon, B. S. 4-1bb (cd137), an inducible costimulatory receptor, as a specific target for cancer therapy. *BMB Rep.* **47**, 122 (2014). 10.5483/BMBRep.2014.47.3.283 PMC4163883 24499671

[21] Kim, A., Nemeth, M. R. & Lim, S.-O. 4–1bb: A promising target for cancer immunotherapy. *Front. Oncol.* **12**, 968360 (2022). 36185242 10.3389/fonc.2022.968360 PMC9515902

[22] Leitner, J., Egerer, R., Waidhofer-Söllner, P., Grabmeier-Pfistershammer, K. & Steinberger, P. Fcr requirements and costimulatory capacity of urelumab, utomilumab, and varlilumab. *Front. Immunol.* **14**, 1208631 (2023). 37575254 10.3389/fimmu.2023.1208631 PMC10413977

[23] Hinner, M. J. et al. Tumor-localized costimulatory t-cell engagement by the 4–1bb/her2 bispecific antibody-anticalin fusion prs-343. *Clin. Cancer Res.* **25**, 5878–5889 (2019). 31138587 10.1158/1078-0432.CCR-18-3654

[24] Shen, A. et al. A novel 4–1bb/her2 bispecific antibody shows potent antitumor activities by increasing and activating tumor-infiltrating t cells. *Am. J. Cancer Res.* **13**, 3246 (2023). 37559991 PMC10408481

[25] Muik, A. et al. Preclinical characterization and phase i trial results of a bispecific antibody targeting pd-l1 and 4–1bb (gen1046) in patients with advanced refractory solid tumors. *Cancer Discov.* **12**, 1248–1265 (2022). 35176764 10.1158/2159-8290.CD-21-1345 PMC9662884

[26] Jeong, S. et al. Novel anti-4-1bb pd-l1 bispecific antibody augments anti-tumor immunity through tumor-directed t-cell activation and checkpoint blockade. *J. Immunother. Cancer* **9** (2021). 10.1136/jitc-2021-002428 PMC8261887 34230109

[27] Montagne, J. M. et al. Cd137 agonism enhances anti-pd1 induced activation of expanded cd8+ t cell clones in a neoadjuvant pancreatic cancer clinical trial. *iScience* **28** (2025). 10.1016/j.isci.2024.111569 PMC11730579 39811671

[28] Trüb, M. et al. Fibroblast activation protein-targeted-4-1bb ligand agonist amplifies effector functions of intratumoral t cells in human cancer. *J. Immunother. Cancer* **8** (2020). 10.1136/jitc-2019-000238 PMC7333869 32616554

[29] Zhai, T. et al. Generation of a safe and efficacious llama single-domain antibody fragment (vhh) targeting the membrane-proximal region of 4-1bb for engineering therapeutic bispecific antibodies for cancer. *J. ImmunoTher. Cancer* **9** (2021). 10.1136/jitc-2020-002131 PMC8237747 34172514

[30] Birtalan, S. et al. The intrinsic contributions of tyrosine, serine, glycine and arginine to the affinity and specificity of antibodies. *J. Mol. Biol.* **377**, 1518–1528 (2008). 18336836 10.1016/j.jmb.2008.01.093

[31] Conte, L. L., Chothia, C. & Janin, J. The atomic structure of protein-protein recognition sites. *J. Mol. Biol.* **285**, 2177–2198 (1999). 9925793 10.1006/jmbi.1998.2439

[32] Zemlin, M. et al. Expressed murine and human cdr-h3 intervals of equal length exhibit distinct repertoires that differ in their amino acid composition and predicted range of structures. *J. Mol. Biol.* **334**, 733–749 (2003). 14636599 10.1016/j.jmb.2003.10.007

[33] Khass, M., Vale, A. M., Burrows, P. D. & Schroeder, H. W. Jr. The sequences encoded by immunoglobulin diversity (dh) gene segments play key roles in controlling b-cell development, antigen-binding site diversity, and antibody production. *Immunol. Rev.* **284**, 106–119 (2018). 29944758 10.1111/imr.12669

[34] Davies, D. R. & Cohen, G. H. Interactions of protein antigens with antibodies. *Proc. Natl. Acad. Sci.* **93**, 7–12 (1996). 8552677 10.1073/pnas.93.1.7 PMC40169

[35] Mejias-Gomez, O. et al. A window into the human immune system: comprehensive characterization of the complexity of antibody complementary-determining regions in functional antibodies. In *MAbs*, vol. 15, 2268255 (Taylor & Francis, 2023). 10.1080/19420862.2023.2268255 PMC10601506 37876265

[36] Madsen, A. V. et al. Structural trends in antibody-antigen binding interfaces: a computational analysis of 1833 experimentally determined 3d structures. *Comput. Struct. Biotechnol. J.* **23**, 199–211 (2024). 38161735 10.1016/j.csbj.2023.11.056 PMC10755492

[37] Jiang, J. et al. Sars-cov-2 antibodies recognize 23 distinct epitopic sites on the receptor binding domain. *Commun. Biol.* **6**, 953 (2023). 37726484 10.1038/s42003-023-05332-w PMC10509263

[38] Prassler, J. et al. Hucal platinum, a synthetic fab library optimized for sequence diversity and superior performance in mammalian expression systems. *J. Mol. Biol.* **413**, 261–278 (2011). 21856311 10.1016/j.jmb.2011.08.012

[39] Fellouse, F. A. et al. High-throughput generation of synthetic antibodies from highly functional minimalist phage-displayed libraries. *J. Mol. Biol.* **373**, 924–940 (2007). 17825836 10.1016/j.jmb.2007.08.005

[40] Altschul, S. F., Gish, W., Miller, W., Myers, E. W. & Lipman, D. J. Basic local alignment search tool. *J. Mol. Biol.* **215**, 403–410 (1990). 2231712 10.1016/S0022-2836(05)80360-2

[41] Bennett, N. R. et al. Atomically accurate de novo design of antibodies with rfdiffusion. *bioRxiv*10.1101/2024.03.14.585103 (2025). https://www.biorxiv.org/content/early/2025/02/28/2024.03.14.585103.full.pdf. 10.1038/s41586-025-09721-5 PMC12727541 41193805

[42] Jumper, J. et al. Highly accurate protein structure prediction with alphafold. *Nature* **596**, 583–589 (2021). 34265844 10.1038/s41586-021-03819-2 PMC8371605

[43] Polonsky, K., Pupko, T. & Freund, N. T. Evaluation of the ability of alphafold to predict the three-dimensional structures of antibodies and epitopes. *J. Immunol.* **211**, 1578–1588 (2023). 37782047 10.4049/jimmunol.2300150

[44] Zarschler, K., Witecy, S., Kapplusch, F., Foerster, C. & Stephan, H. High-yield production of functional soluble single-domain antibodies in the cytoplasm of escherichia coli. *Microb. Cell Fact.* **12**, 1–13 (2013). 24161153 10.1186/1475-2859-12-97 PMC3818982

[45] Ma, L. et al. Preclinical development of a novel cd47 nanobody with less toxicity and enhanced anti-cancer therapeutic potential. *J. Nanobiotechnol.* **18**, 1–15 (2020). 10.1186/s12951-020-0571-2 PMC6956557 31931812

[46] Gonzalez-Perez, D. et al. Random and combinatorial mutagenesis for improved total production of secretory target protein in escherichia coli. *Sci. Rep.* **11**, 5290 (2021). 33674702 10.1038/s41598-021-84859-6 PMC7935960

[47] Yao, D., Su, L., Li, N. & Wu, J. Enhanced extracellular expression of bacillus stearothermophilus -amylase in bacillus subtilis through signal peptide optimization, chaperone overexpression and -amylase mutant selection. *Microb. Cell Fact.* **18**, 1–12 (2019). 30971250 10.1186/s12934-019-1119-8 PMC6458788

[48] Ni, Y. & Chen, R. Extracellular recombinant protein production from escherichia coli. *Biotech. Lett.* **31**, 1661–1670 (2009). 10.1007/s10529-009-0077-3 19597765

[49] Bond, C. J., Marsters, J. C. Jr. & Sidhu, S. S. Contributions of cdr3 to vhh domain stability and the design of monobody scaffolds for naive antibody libraries. *J. Mol. Biol.* **332**, 643–655 (2003). 12963373 10.1016/s0022-2836(03)00967-7

[50] Tohidkia, M. R., Sepehri, M., Khajeh, S., Barar, J. & Omidi, Y. Improved soluble scfv elisa screening approach for antibody discovery using phage display technology. *SLAS DISCOVERY: Advancing Life Sciences R &D* **22**, 1026–1034 (2017). 28346811 10.1177/2472555217701059

[51] Murakami, T. et al. Construction of a humanized artificial vhh library reproducing structural features of camelid vhhs for therapeutics. *Antibodies* **11**, 10 (2022). 35225868 10.3390/antib11010010 PMC8884020

[52] Rouet, R., Dudgeon, K., Christie, M., Langley, D. & Christ, D. Fully human vh single domains that rival the stability and cleft recognition of camelid antibodies. *J. Biol. Chem.* **290**, 11905–11917 (2015). 25737448 10.1074/jbc.M114.614842 PMC4424330

[53] Eliseev, I. E. et al. Targeting erbb3 receptor in cancer with inhibitory antibodies from llama. *Biomedicines* **9**, 1106 (2021). 34572289 10.3390/biomedicines9091106 PMC8467012

[54] Eliseev, I. E. et al. Crystal structures of a llama vhh antibody bcd090-m2 targeting human erbb3 receptor. *F1000Research* **7** (2018). 10.12688/f1000research.13612.1 PMC6097396 30430004

[55] Kabat, E. A. *Sequences of proteins of immunological interest*. 91 (US Department of Health and Human Services, Public Health Service, National, 1991).

[56] Huang, P.-S. et al. Rosettaremodel: a generalized framework for flexible backbone protein design. *PLoS ONE* **6**, e24109 (2011). 21909381 10.1371/journal.pone.0024109 PMC3166072

[57] Chin, S. M. et al. Structure of the 4–1bb/4-1bbl complex and distinct binding and functional properties of utomilumab and urelumab. *Nat. Commun.* **9**, 4679 (2018). 30410017 10.1038/s41467-018-07136-7 PMC6224509

[58] Li, Y. et al. Limited cross-linking of 4–1bb by 4–1bb ligand and the agonist monoclonal antibody utomilumab. *Cell Rep.* **25**, 909–920 (2018). 30355497 10.1016/j.celrep.2018.09.073

[59] Ivanisenko, N. V. et al. Sema 2.0: web-platform for b-cell conformational epitopes prediction using artificial intelligence. *Nucleic Acids Res.* **52**, W533–W539 (2024). 10.1093/nar/gkae386 PMC11223818 38742639

[60] Dauparas, J. et al. Robust deep learning-based protein sequence design using proteinmpnn. *Science* **378**, 49–56 (2022). 36108050 10.1126/science.add2187 PMC9997061

[61] DiMaio, F., Leaver-Fay, A., Bradley, P., Baker, D. & André, I. Modeling symmetric macromolecular structures in rosetta3. *PLoS ONE* **6**, e20450 (2011). 21731614 10.1371/journal.pone.0020450 PMC3120754

[62] Bennett, N. R. et al. Improving de novo protein binder design with deep learning. *Nat. Commun.* **14**, 2625 (2023). 37149653 10.1038/s41467-023-38328-5 PMC10163288

[63] Khatib, F. et al. Algorithm discovery by protein folding game players. *Proc. Natl. Acad. Sci.* **108**, 18949–18953 (2011). 22065763 10.1073/pnas.1115898108 PMC3223433

[64] Maguire, J. B. et al. Perturbing the energy landscape for improved packing during computational protein design. *Proteins Struct. Funct. Bioinf.* **89**, 436–449 (2021). 10.1002/prot.26030 PMC8299543 33249652

[65] Shrake, A. & Rupley, J. A. Environment and exposure to solvent of protein atoms. lysozyme and insulin. *J. Mol. Biol.* **79**, 351–371 (1973). 10.1016/0022-2836(73)90011-9 4760134

[66] Miller, N. L., Clark, T., Raman, R. & Sasisekharan, R. Learned features of antibody-antigen binding affinity. *Front. Mol. Biosci.* **10**, 1112738 (2023). 36895805 10.3389/fmolb.2023.1112738 PMC9989197

