# Supplementary material for: An iterative strategy to design 4-1BB agonist nanobodies de novo with generative AI models
Source: Sci Rep. 2025 Jul 14;15:25412. doi: 10.1038/s41598-025-10241-5 (PMC12259950; doi:10.1038/s41598-025-10241-5)
Supplement: Supplementary file 2 — Supplementary Information 2. [file 41598_2025_10241_MOESM2_ESM.pdf]

An Iterative Strategy to Design 4-1BB Agonist Nanobodies De Novo with Generative AI Models

Ivan Poddiakov[1,+,*], Dmitriy Umerenkov[2,+], Irina Shulcheva[4,+], Victoria Golovina[3], Vasilina Borisova[3,5], Irina Pozdnyakova-Filatova[3,6], Evgeniy Loktyushov[4], Galina Zubkova[1], Andrey Savchenko[1,7], Andrei Ulitin[4,], and Pavel Blinov[1]**

[1]Sber AI Lab, Moscow, Russia
[2]AIRI, Moscow, Russia
[3]BigBioBang LLC, Pushchino, Moscow Region, Russia
[4]Pushchino Scientific Center for Biological Research of the Russian Academy of Sciences, Institute for Biological Instrumentation, Pushchino, Moscow Region, Russia
[5]MIREA — Russian Technological University, Moscow, Russia
[6]Pushchino Scientific Center for Biological Research of the Russian Academy of Sciences, G.K. Skryabin Institute of Biochemistry and Physiology of Microorganisms, Pushchino, Moscow Region, Russia
[7]Laboratory of Algorithms and Technologies for Network Analysis, HSE University, Nizhny Novgorod, Russia
[+]These authors contributed equally
[*]Correspondence: ivanpodd@gmail.com
[**]Correspondence: 1974snail@gmail.com

Supplementary figures

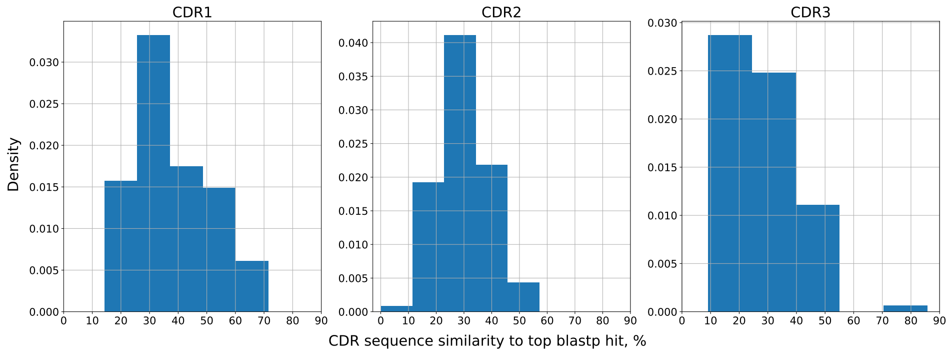

Supplementary Figure 1. CDR sequences similarity with top blastp hit for each Step 2 candidate. X-axis represents CDR sequence similarity to analogous CDR of the top blast hit.

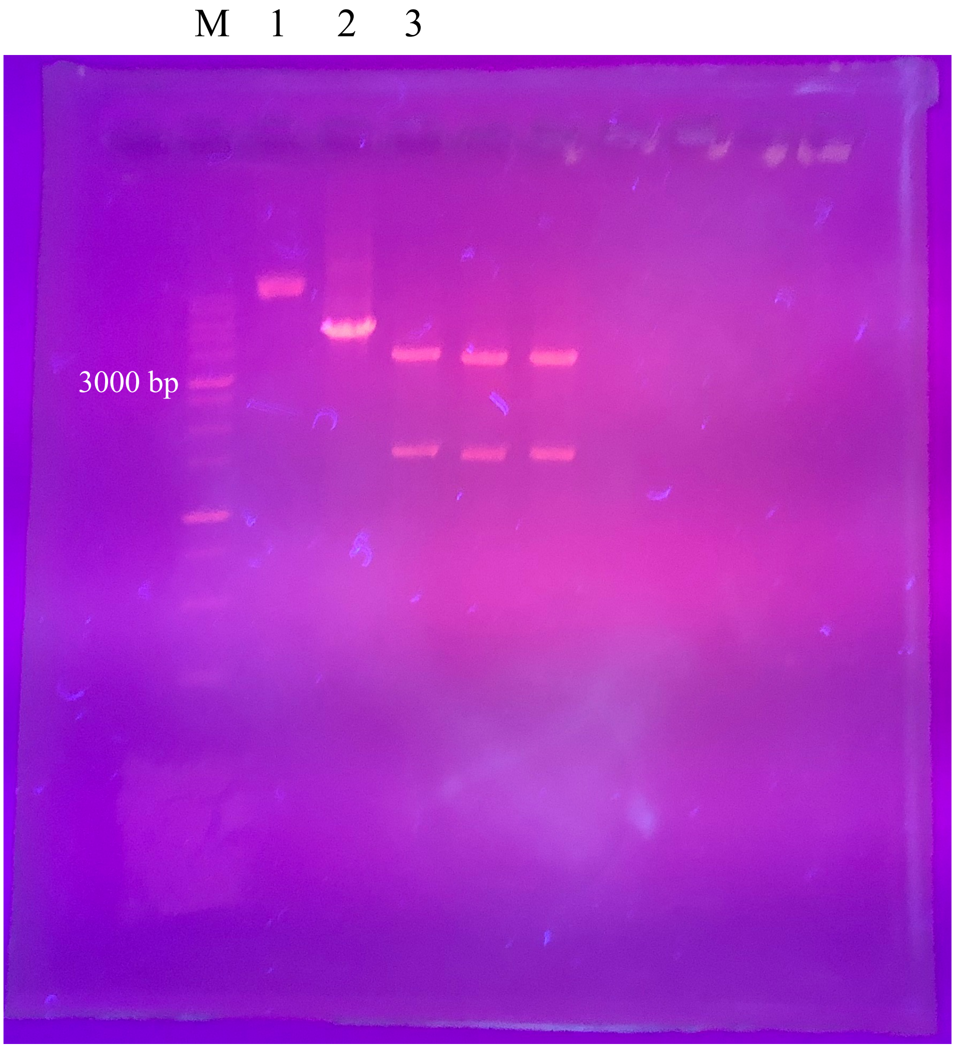

Supplementary Figure 2. Typical experiment electropherogram.
Electropherogram of the pET22b Plasmid Vector in a 1% Agarose Gel. 1 – Purified pET22b vector; 2 – pET22b after inverse PCR; 3 – pET22b after digestion with the BsaI restriction enzyme. M – DNA ladder 1kbp+ (Eurogen, Russia).

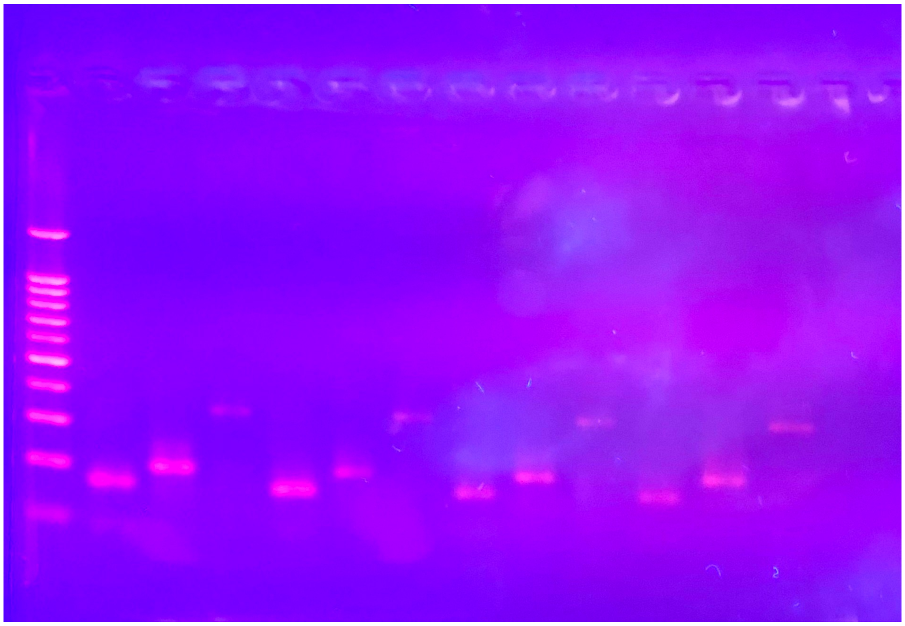

Supplementary Figure 3. Typical experiment electropherogram.
Electropherogram of PCR Products for VHH Gene Assembly in a 2% Agarose Gel. 1 – First half of the gene (160 bp); 2 – Second half of the gene (180 bp); 3 – Full-length VHH gene after splice PCR reaction (310 bp). M – DNA ladder 100bp+ (Eurogen, Russia).

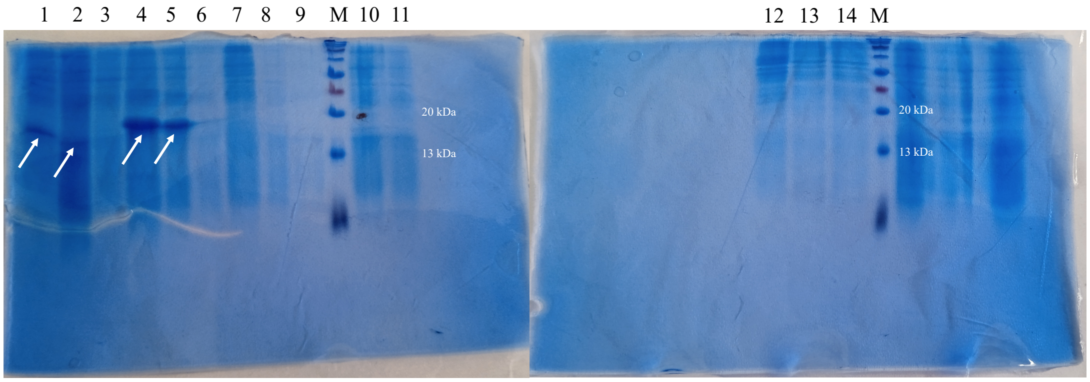

Supplementary Figure 4. Typical electropherogram in 20% PAAG of the analysis of culture media after expression of the VHH protein.
1 – control protein VHH 7D4B after expression, 2 – clone 7-1, 3 – clone 28-1, 4 – clone 31-1, 5 – clone 31-2, 6 – clone 33-1, 7 – clone 33-2, 8 – clone 35-1, 9 – clone 35-2, M – molecular weight marker of proteins, 10 – clone 36-1, 11 – clone 36-2, 12 – clone 40-2, 13 – clone 26-1, 14 – clone 26-2. The arrows indicate the target product.

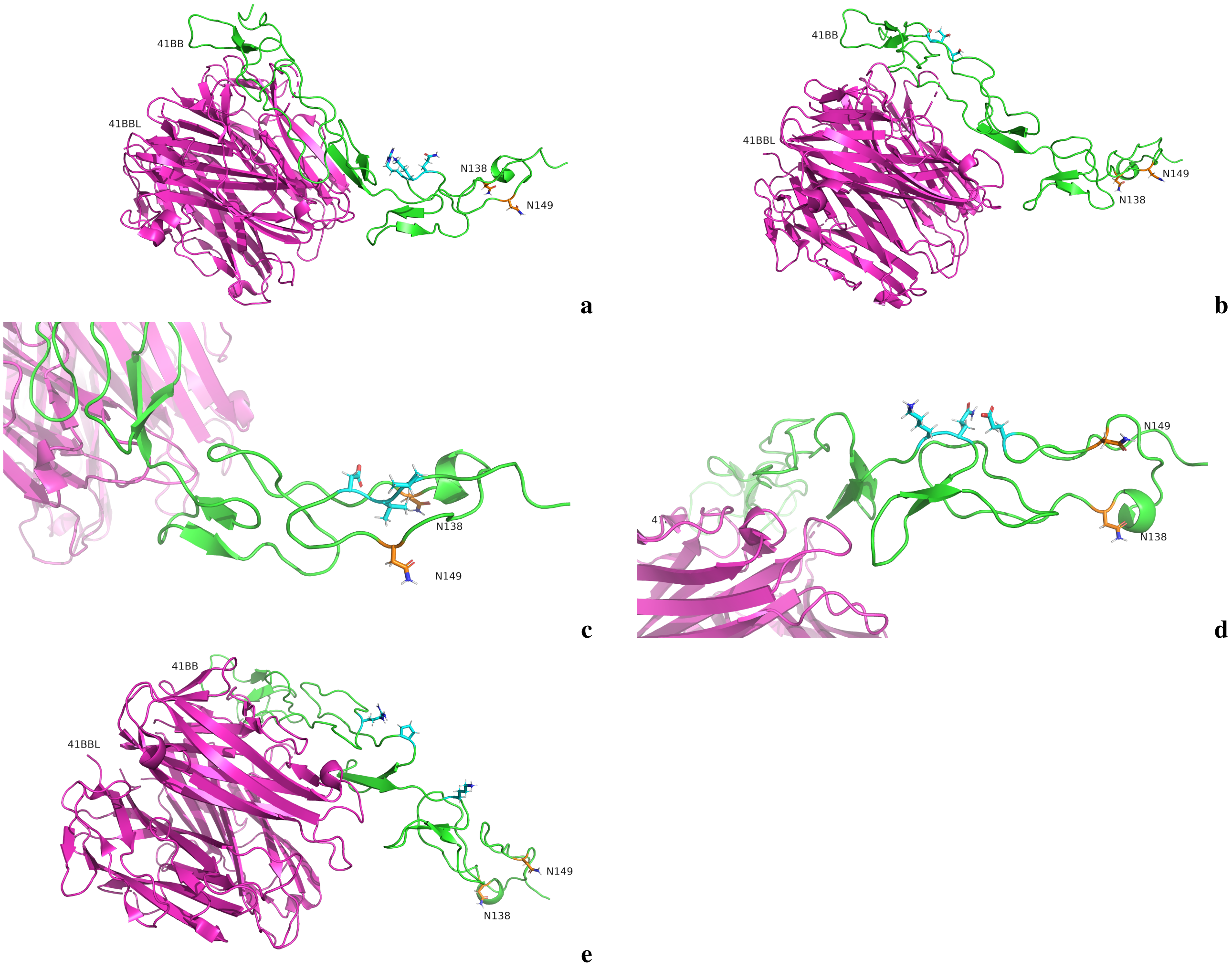

Supplementary Figure 5. 4-1BB surface availability and selected epitopes.
A significant part of 4-1BB (green) engages in interaction with its ligand 4-1BBL (magenta); its N138 and N149 (orange) undergo post-translational modifications. **a** - epitope 1: Q128, K129 and R130 highlighted in blue, **b** - epitope 2: S80, T81 and S82; **c** - epitope 3: D155, V156 and V157; **d** - epitope 4: K107, Q108 and E153; **e** - epitope 5: R73, P90 and K107.

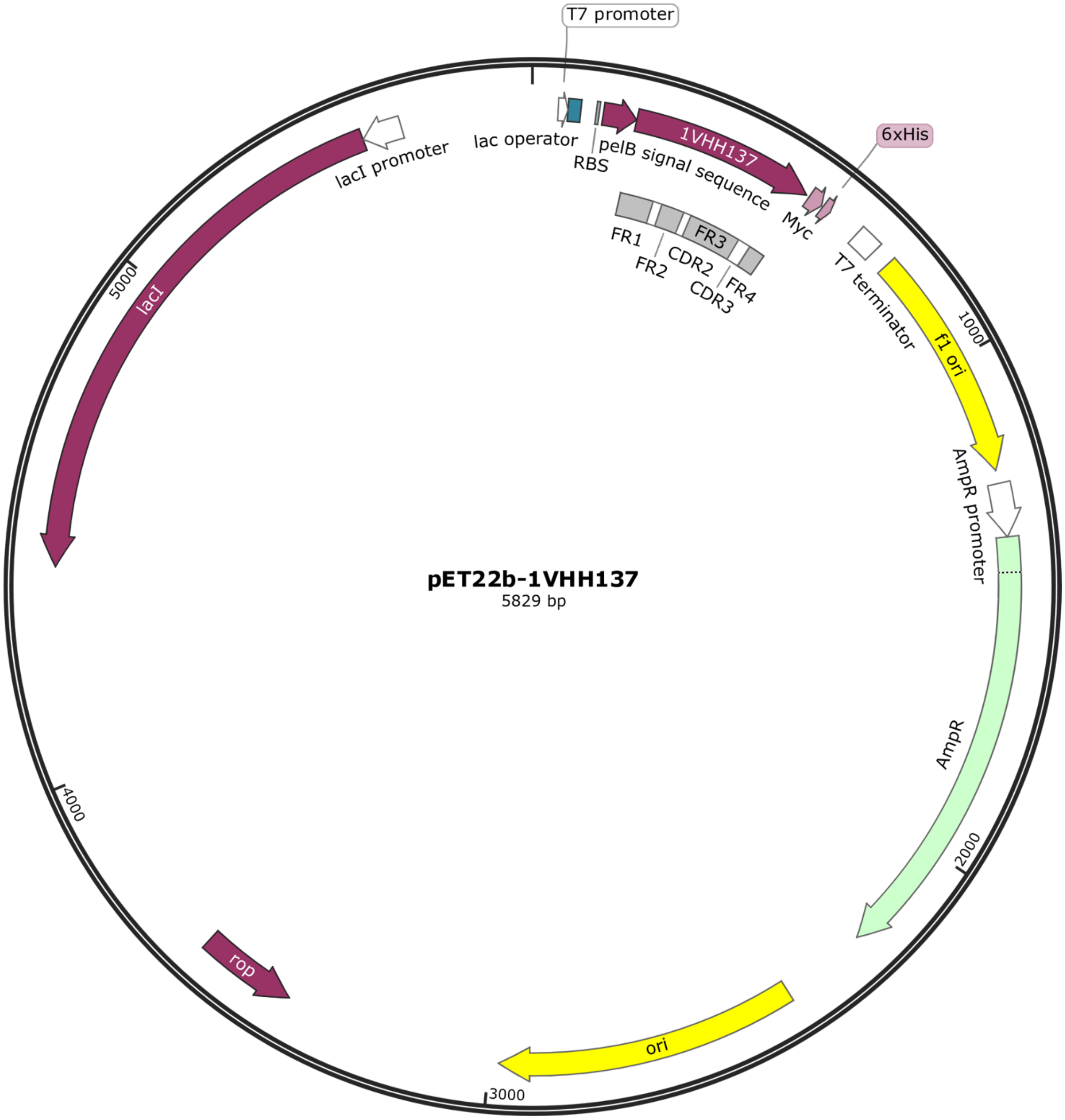

Supplementary Figure 6. The map of the pET22b-VHH vector.
pET22b-VHH vector map, with the VHH gene, followed by myc-tag and his-tag under T7 promoter.

Supplementary Tables

Supplementary Table 1. Key computational parameters.

Parameter	Value
RFdiffusion steps	50
RFdiffusion checkpoint	Complex_base_ckpt.pt
Fixed residues (Kabat numbering)	1–26, 34–52, 58–95, 100–113
Residues to generate for CDRs 1, 2 and 3	7, 7, 6-12
ProteinMPNN checkpoint	v_48_020.pt
ProteinMPNN cycles in Step 1	2
Sequence per backbone per cycle in Step 1	1
ProteinMPNN sampling T in Step 1	$10^{-}6$
ProteinMPNN cycles in Step 2	up to 50 * 16
Sequence per backbone per cycle in Step 2	1
ProteinMPNN sampling T in Step 2	0.25
AlphaFold2 checkpoint	alphafold_params_2022-12-06
